# The Evaluation of Multiple Linear Regression–Based Limited Sampling Strategies for Mycophenolic Acid in Children with Nephrotic Syndrome

**DOI:** 10.3390/molecules26123723

**Published:** 2021-06-18

**Authors:** Joanna Sobiak, Matylda Resztak, Maria Chrzanowska, Jacek Zachwieja, Danuta Ostalska-Nowicka

**Affiliations:** 1 Department of Physical Pharmacy and Pharmacokinetics, Poznan University of Medical Sciences, 60-781 Poznań, Poland; mresztak@ump.edu.pl (M.R.); mchrzan@ump.edu.pl (M.C.); 2 Department of Pediatric Nephrology and Hypertension, Poznan University of Medical Sciences, 60-572 Poznań, Poland; zachwiej@mp.pl (J.Z.); dostalska@ump.edu.pl (D.O.-N.)

**Keywords:** mycophenolate mofetil, mycophenolic acid, pediatric patients, limited sampling strategy, multiple linear regression, therapeutic drug monitoring

## Abstract

We evaluated mycophenolic acid (MPA) limited sampling strategies (LSSs) established using multiple linear regression (MLR) in children with nephrotic syndrome treated with mycophenolate mofetil (MMF). MLR-LSS is an easy-to-determine approach of therapeutic drug monitoring (TDM). We assessed the practicability of different LSSs for the estimation of MPA exposure as well as the optimal time points for MPA TDM. The literature search returned 29 studies dated 1998–2020. We applied 53 LSSs (*n* = 48 for MPA, *n* = 5 for free MPA [fMPA]) to predict the area under the time-concentration curve (AUC_pred_) in 24 children with nephrotic syndrome, for whom we previously determined MPA and fMPA concentrations, and compare the results with the determined AUC (AUC_total_). Nine equations met the requirements for bias and precision ±15%. The MPA AUC in children with nephrotic syndrome was predicted the best by four time-point LSSs developed for renal transplant recipients. Out of five LSSs evaluated for fMPA, none fulfilled the ±15% criteria for bias and precision probably due to very high percentage of bound MPA (99.64%). MPA LSS for children with nephrotic syndrome should include blood samples collected 1 h, 2 h and near the second MPA maximum concentration. MPA concentrations determined with the high performance liquid chromatography after multiplying by 1.175 may be used in LSSs based on MPA concentrations determined with the immunoassay technique. MPA LSS may facilitate TDM in the case of MMF, however, more studies on fMPA LSS are required for children with nephrotic syndrome.

## 1. Introduction

Mycophenolate mofetil (MMF) is an immunosuppressive drug administered in the prophylaxis against acute rejection after solid organ transplantation as well as in autoimmune diseases [1], nephrotic syndrome [2,3], and atopic dermatitis [4]. The MMF active moiety, mycophenolic acid (MPA), is characterized by complex and variable pharmacokinetics and high serum albumin binding (97–99%) [1,5]. MPA pharmacokinetics in renal transplant recipients are widely described in the literature [1,6,7,8,9,10], however, although the pharmacokinetics are assumed to be different, there are few studies concerning children with nephrotic syndrome treated with MMF [11,12,13,14]. In our previous study [11], we observed that the target values of the pharmacokinetic parameters, such as the concentration before the next dose (C_0_) and the area under the concentration—time curve from 0 to 12 h (AUC_total_), in children with nephrotic syndrome treated with MMF should be higher than those recommended after renal transplantation [1]. Similar observations were described by other authors [12,15].

MPA therapeutic drug monitoring (TDM) is frequently recommended, mainly to avoid underexposure [1,16]. TDM was shown to be favorable not only in renal transplant recipients [6], but also in patients with lupus nephritis [17] and steroid-dependent nephrotic syndrome [12,13]. One method of TDM is the limited sampling strategy (LSS), which allows us to predict AUC_total_ on the basis of only few blood samples [6] instead of the time-consuming, expensive, and uncomfortable to patients method of collecting 8 to 15 blood samples over 12 h for a full pharmacokinetic profile [18]. LSS may be calculated using the Bayesian approach or multiple linear regression (MLR) analysis, which uses an equation derived from stepwise regression analysis based on concentrations measured at pre-defined times after dosing [16,19]. MLR is easier to use than Bayesian analysis, although one important limitation of the MLR approach is the reliance of the equations on the accuracy of the exact times of blood sample collection [7,16]. MLR LSSs have been proposed for MPA in many groups of patients [8,9,20,21]. Whereas many authors emphasize that each LSS should be applied to the same group of patients as it was established [22], Ting et al. [20] observed that the application of LSSs established for lung transplant recipients to the heart transplant population yielded satisfactory prediction results, Gellermann et al. [15] applied the LSSs established for children after renal transplantation and adult heart transplant recipients to evaluate AUC in children with nephrotic syndrome, and Katsuno et al. [17] used the LSS established for renal transplant recipients to predict AUC in patients with lupus nephritis. Additionally, Tong et al. [23] applied the LSS established with the high performance liquid chromatography (HPLC) method to evaluate the AUC for patients for whom the enzyme multiplied immunoassay technique (EMIT) was used for MPA determination, while Neuberger et al. [24] applied an MPA LSS established after the administration of another MPA formulation, enteric-coated mycophenolic sodium (EC-MPS), in MMF treated patients.

Due to the small number of studies on MPA pharmacokinetics in children with nephrotic syndrome, in this study we evaluated MLR-based LSSs found in the literature in children with nephrotic syndrome treated with MMF. The evaluation aimed to assess the practicability of different LSSs for the estimation of MPA exposure as well as to find the optimal time points for MPA TDM.

## 2. Results

### 2.1. MPA and fMPA Pharmacokinetics

**Figure 1:**
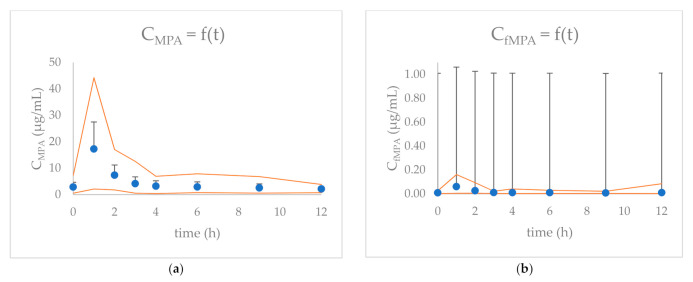
The concentration (+SD) versus time graphs for: (**a**) MPA and (**b**) fMPA for 24 children included in the study. Orange curves indicate the maximum and minimum concentrations at each time-point.

The MPA and free MPA (fMPA) concentrations versus time in 24 children with nephrotic syndrome treated with MMF are presented in Figure 1. The results of MPA and fMPA maximum concentration (C_max_), time to reach C_max_ (t_max_), and AUC_total_ values are presented in Table 1. MPA C_0_ was above 2.0 μg/mL and above 3.0 μg/mL in 67% (*n* = 16) and 42% (*n* = 10) of children, respectively. MPA C_max_ was observed 1 h after MMF administration in 79% of children. Out of 24 children, 63% (*n* = 15) had MPA AUC_total_ within the 30–60 μg∙h/mL range. For 21% (*n* = 5) of children, MPA AUC_total_ was above 60 μg∙h/mL. Mean MPA binding to plasma protein was 99.65%, with only 0.35% of fMPA.

### 2.2. The Evaluation of MLR LSSs in Children with Nephrotic Syndrome

The search of the literature returned 29 studies meeting the requirements concerning MLR LSSs for MPA and fMPA, dated 1998–2020. We applied 48 MPA LSSs [8,9,14,21,22,25,26,27,28,29,30,31,32,33,34,35,36,37,38,39,40,41,42,43,44,45,46,47,48] and five fMPA LSSs [35,36,42] found in the literature to calculate the predicted area under the (0–12 h) time–concentration curve (AUC_pred_) in children with nephrotic syndrome treated with MMF, and compared the results with AUC_total_. In the majority of studies, calcineurin inhibitors (CsA or tacrolimus (Tac)) were co-administered with MMF. In two studies, only MMF was administered and in one other study, only 8% of patients received CsA concomitantly. The majority of studies concerned patients after solid organ transplantation. We found seven studies including pediatric patients after renal transplantation (*n* = 4), with nephrotic syndrome (*n* = 2), and with lupus erythematosus (*n* = 1). In order to better describe the results, we divided the LSSs according to the methods of MPA determination and subdivided according to the indications for MMF treatment (Table 2 and Table 3). The LSSs for fMPA are presented separately (Table 4).

The predictive performances for the estimation of MPA AUC_pred_ using the 23 MPA MLR LSSs available in the literature in which MPA was determined based on HPLC method are presented in Table 2. Only two out of 23 equations (9%) met the requirements of ±15% for %MPE and 15% for %MAE. If the acceptable %MPE and %MAE were extended to ±20%, 13 equations (57%) would fulfill the criteria. For two of the 23 LSSs (9%), AUC_pred_ was within ±15% of AUC_total_ for more than 60% of children, concomitantly with r^2^ above 0.800. These LSSs included C_1_-C_2_-C_4_ and C_1_-C_2_-C_6_, both of which were established for Tac co-administration. High r^2^ was found in the Gota et al. [34] equation, concomitantly with low predictive performance. A number of 11 LSSs (48%) gave an AUC_pred_ within ±15% of the AUC_total_ for less than 50% of children.

The predictive performances of 25 MPA MLR LSSs in which MPA was determined based on EMIT or particle enhanced turbidimetric inhibition immunoassay (PETINIA) are presented in Table 3. Seven of 25 LSSs (28%) met the requirements of ±15% for %MPE and 15% for %MAE. If the acceptable %MPE and %MAE were extended to ±20%, ten equations (40%) would fulfill the criteria. For three of 25 LSSs (12%), the AUC_pred_ was within ±15% of the AUC_total_ for more than 60% of children, concomitantly with r^2^ above 0.800. These LSSs included C_1_-C_2_-C_4_-C_6_ (two LSSs) and C_0_-C_1_-C_3_-C_6,_ all of which were established for Tac co-administration. In 13 of 25 LSSs (52%), the AUC_pred_ was within ±15% of the AUC_total_ in less than 50% of children.

We found five MLR LSSs for fMPA in three studies which we applied to calculate the fMPA AUC_pred_ for children with nephrotic syndrome. The predictive performance of the fMPA MLR LSSs is presented in Table 4. In all three studies, MPA was determined with the HPLC method. None of the equations fulfilled the criteria for %MPE and %MAE. There was one four time point equation (C_1_-C_2_-C_4_-C_6_), which was established for patients after liver transplantation and co-treated with Tac, which met the requirements of ±20% for %MPE and %MAE, and demonstrated an r^2^ above 0.800.

### 2.3. Comparison of the Best Matched MLR LSSs

**Figure 2:**
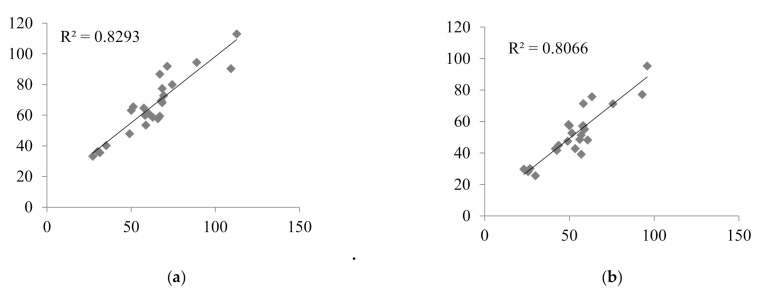

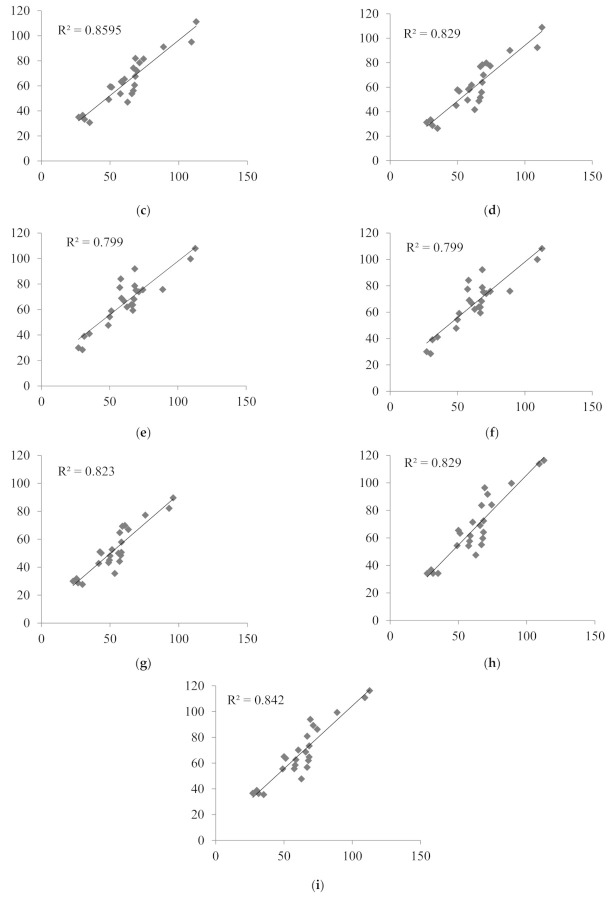
Correlations between the MPA AUC_total_ and the MPA AUC_pred_ calculated for children with nephrotic syndrome using MLR LSS equations found in the literature that fulfilled the criteria for %MPE and %MAE ±15%; (**a**) AUC_pred_ = 7.4 + 2.3 × C_0_ + 1.2 × C_1_ + 2.3 × C_3_ + 4.4 × C_6_ [30]; (**b**) AUC_pred_ = 9.328 + 1.311 × C_1_ + 1.455 × C_2_ + 2.901 × C_4_ [43]; (**c**) AUC_pred_ = 10.6 + 1.1 × C_1_ + 1.1 × C_2_ + 2.0 × C_4_ + 3.9 × C_6_ [30]; (**d**) AUC_pred_ = 5.92 + 1.10 × C_1_ + 1.01 × C_2_ + 1.77 × C_4_ + 4.80 × C_6_ [28]; (**e**) AUC_pred_ = 8.22 + 3.16 × C_0_ + 0.99 × C_1_ + 1.33 × C_2_ + 4.18 × C_4_ [32]; (**f**) AUC_pred_ = 8.217 + 3.163 × C_0_ + 0.994 × C_1_ + 1.334 × C_2_ + 4.183 × C_4_ [8]; (**g**) AUC_pred_ = 10.229 + 0.925 × C_1_ + 1.750 × C_2_ + 4.586 × C_6_ [29]; (**h**) AUC_pred_ = 7.73 + 0.94 × C_1_ + 2.55 × C_2_ + 5.48 × C_6_ [32]; (**i**) AUC_pred_ = 10.75 + 0.98 × C_1_ + 2.38 × C_2_ + 4.86 × C_6_ [33].

**Figure 3:**
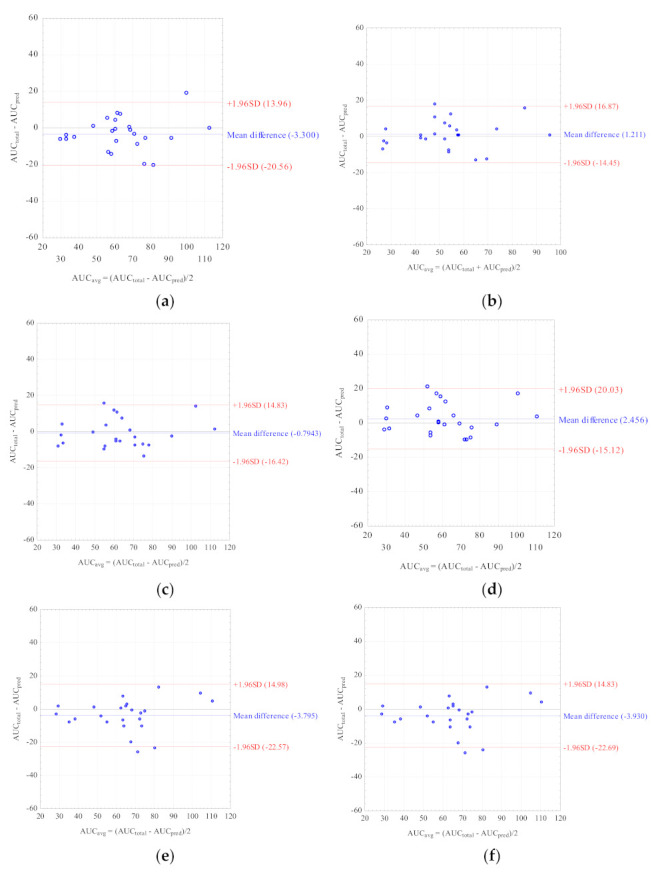

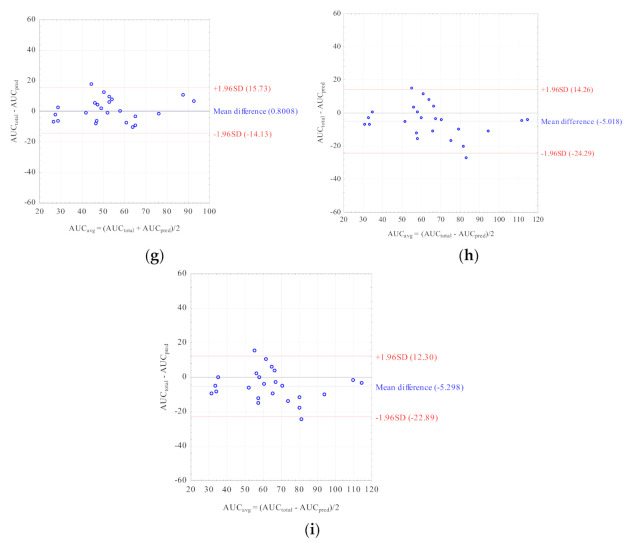
Bland–Altman analyses testing agreement between the MPA AUC_total_ and the MPA AUC_pred_ calculated for children with nephrotic syndrome using the MLR LSS equations found in the literature that fulfilled the criteria for %MPE and %MAE ± 15%; (**a**) AUC_pred_ = 7.4 + 2.3 × C_0_ + 1.2 × C_1_ + 2.3 × C_3_ + 4.4 × C_6_ [30]; (**b**) AUC_pred_ = 9.328 + 1.311 × C_1_ + 1.455 × C_2_ + 2.901 × C_4_ [43]; (**c**) AUC_pred_ = 10.6 + 1.1 × C_1_ + 1.1 × C_2_ + 2.0 × C_4_ + 3.9 × C_6_ [30]; (**d**) AUC_pred_ = 5.92 + 1.10 × C_1_ + 1.01 × C_2_ + 1.77 × C_4_ + 4.80 × C_6_ [28]; (**e**) AUC_pred_ = 8.22 + 3.16 × C_0_ + 0.99 × C_1_ + 1.33 × C_2_ + 4.18 × C_4_ [32]; (**f**) AUC_pred_ = 8.217 + 3.163 × C_0_ + 0.994 × C_1_ + 1.334 × C_2_ + 4.183 × C_4_ [8]; (**g**) AUC_pred_ = 10.229 + 0.925 × C_1_ + 1.750 × C_2_ + 4.586 × C_6_ [29]; (**h**) AUC_pred_ = 7.73 + 0.94 × C_1_ + 2.55 × C_2_ + 5.48 × C_6_ [32]; (**i**) AUC_pred_ = 10.75 + 0.98 × C_1_ + 2.38 × C_2_ + 4.86 × C_6_ [33].

Nine LSSs with %MPE and %MAE ±15%, and r^2^ ≥ 0.799 were considered the best. These equations were established for adult renal transplant recipients (*n* = 3), adult liver transplant recipients (*n* = 2), and pediatric renal transplant recipients (*n* = 4). For these equations, the graphs describing the correlations between the AUC_total_ and the AUC_pred_ were drawn (Figure 2), and Bland–Altman (Figure 3) tests were performed. For the majority of equations, the Bland–Altman test showed only one or two values exceeding the fixed range of the mean ± 1.96 SD, which confirmed the agreement between the AUC_total_ and the AUC_pred_.

## 3. Discussion

Estimating LSS is the approach of TDM applied for many drugs, e.g., MPA, levofloxacin, and etoposide [49,50,51]. We recently established and compared LSS for MPA in children with nephrotic syndrome using two different approaches [52]. In the present study, we used the MPA LSSs found in the literature in the attempt to assess their practicability for the estimation of MPA exposure and to find the optimal time points for MPA TDM in children with nephrotic syndrome. We verified the LSSs established for different indications, as in the literature we found studies in which LSS developed for one population was used to evaluate LSS in other population [20,24].

The novelty of our study is that we converted MPA concentrations determined with HPLC to evaluate the MPA LSSs established for EMIT or PETINIA. As MPA concentrations are 15–20% higher when established with EMIT or PETINIA due to MPA cross reaction with the MPA metabolite acyl-glucuronide [16,53], we multiplied the HPLC determined concentration by 1.175. Tong et al. [23] used MPA LSSs established for adult heart transplant recipients with the HPLC method to predict the AUC in children with nephrotic syndrome for whom MPA concentrations were determined with EMIT without any adjustment. Our results of predictive performance for both HPLC and EMIT/PETINIA did not differ significantly, and therefore we concluded that this approach may enable using LSSs established with EMIT or PETINIA to predict the MPA AUC based on HPLC-determined concentrations.

Nine MPA LSSs fulfilled the criteria of the best predictive performance. Because MMF is mainly administered as an acute rejection prophylaxis after renal transplantation and most of the studies concerned adults, five out of nine the best MLR LSSs were established for adults [28,29,30,43]. Four LSSs considered as the best were established for pediatric patients [8,32,33]. Among these four LSSs, although two equations were very similar, they were published in two different articles, and we therefore evaluated both of them [8,32]. Seven of nine LSSs included renal transplant recipients, both adult (*n* = 3) [30,43] and pediatric (*n* = 4) [8,32,33]. Two of nine the best LSSs included liver transplant recipients [28,29]. Surprisingly, the LSSs established for children with nephrotic syndrome [14,25] or lupus erythematosus [21] performed poorly as they did not fulfill the criteria: the values of r^2^ were below 0.800, and ≤50% of the AUC_pred_ values were within ±15% of the AUC_total_. These poor results may be explained by one time point equation in the Hibino et al. study [14] and the relatively high intercept.

In our opinion, in the case of MPA, accurate and precise LSSs should consist of at least three time points. Among the best LSSs, four and five LSSs included four and three time points, respectively. The predictive performance for one and two time point LSSs were unsatisfactory. If the criteria were extended to ±20% for %MPE and %MAE, only one two-time-point equation would have fulfilled the criteria. However, the percentage of AUC_pred_ within ±15% of AUC_total_ was rather poor for this equation (50%). Moreover, equations with only one time-point performed poorly with respect to the percentage of the AUC_pred_ within ±15% of the AUC_total_ (≤33%). Interestingly, for one LSS, which included AUC_1–4_ instead of concentration at defined time points [34], r^2^ was >0.800, while the predictive performance and the percentage of the AUC_pred_ within ±15% of the AUC_total_ were unsatisfactory. Moreover, the LSSs which included logarithmic concentrations did not perform well [44].

The inclusion of particular time points may be of significant importance as they reflect MPA pharmacokinetics. In our study, eight of the nine (89%) best-matched equations included C_1_ and C_2,_ and six equations included C_6_. Those three time points coincide with the MPA C_max_ (1–2 h after dosing) and the second maximum concentration (C_max2_; 6–12 h after MMF administration) [10]. This evidence suggests that the MPA C_max_ and C_max2_ influence its AUC the most, and the blood samples should be collected at least in three time points near C_max_ and C_max2_ to precisely predict the AUC. According to the literature, for children with nephrotic syndrome C_2_ or time points up to 2 h after MMF administration should be included in the MPA LSS [14,25]. The inclusion of C_6_ makes using LSS cumbersome. However, according to the literature, better predictive performance was observed for LSSs which included time points in the latter half of the dosing interval [16]. Out of the nine best matched equations, only 3 (33%) included C_0_. This observation is in accordance with the literature data, as MPA C_0_ correlates poorly with AUC_total_ [6].

We evaluated the MLR LSSs found in the literature regardless the drugs co-administered with MMF. Five of nine the best LSSs were established for MMF- and Tac-treated patients. According to the literature, Tac does not influence MPA clearance [3], and in patients with autoimmune disease MPA clearance is likely to be in close agreement with estimates from renal allograft recipients co-treated with Tac [54]. On the other hand, MPA concentrations are lower if co-administered with CsA [10]. CsA inhibits MPA enterohepatic recirculation, causing a decrease in MPA exposition, and therefore blood sampling does not require including time-points around the MPA C_max2_ when MMF is co-administered with CsA [16]. Among the LSSs applied in this study, only in three studies with MLR LSSs [21,25,46] did the patients not receive concomitant medications (in one study only 8% of patients received CsA [46]). Surprisingly, in our study, for these LSSs the predictive performance fell beyond ±15% range. The equation from the Prabha et al. [21] study would have fulfilled the extended criteria (±20%). One equation, which included C_6_, from the de Winter et al. study [46], was characterized by the r^2^ being >0.800, however, it did not fulfill even the extended criteria, therefore, we confirmed that choosing model equations based only on their r^2^ values may be misleading [55].

Out of five LSSs developed for fMPA [35,36,42], none fulfilled the criteria when used to evaluate the fMPA AUC_pred_ in children with nephrotic syndrome. One equation would fulfill the criteria extended to ±20%, but the percentage of the AUC_pred_ within ±15% of the AUC_total_ for this formula was poor (38%). The obtained results may indicate differences in MPA protein binding in children with nephrotic syndrome. According to the literature, MPA is bound to plasma proteins in 97% to 99% [29,56]. In our previous study [11], similarly as in this study, the median fMPA fraction was 0.36%, which gives very high percentage of bound MPA (99.64%).

The limitation of our study is the fact that we were unable to apply the LSSs with time points 0.5, 0.75, or 1.5 h after MMF administration as blood sampling was not so frequent in the children included in the study.

## 4. Materials and Methods

### 4.1. Ethical Considerations

The study was approved by the Bioethical Committee at Poznan University of Medical Sciences and it is in accordance with the 1964 Declaration of Helsinki and its later amendments. Informed consent was obtained from the parents or guardians prior to initiating the study.

### 4.2. Children’s Characteristics

Our study included 24 children, aged 3–18 years, with nephrotic syndrome treated with MMF and steroids in the Department of Pediatric Nephrology and Hypertension, Poznan University of Medical Sciences, Poland. MMF was administered orally twice a day at the same dose. On the day of blood collection, 18 children were in remission whereas six children had trace proteinuria. MMF was given under fasting conditions, 30 min before breakfast. The exclusion criteria were cyclosporine (CsA) co-administration, MMF dosing at unequal morning and evening doses, administration of MMF shorter than 1 month and too low number of blood samples. Blood samples were collected into EDTA tubes before MMF administration (C_0_) and subsequently 1 h (C_1_), 2 h (C_2_), 3 h (C_3_), 4 h (C_4_), 6 h (C_6_), 9 h (C_9_), and 12 h (C_12_) after its administration. The samples were centrifuged to obtain plasma, then immediately frozen and kept at −20 °C until analysis. The demographic and biochemical characteristics of the children are presented in Table 5.

### 4.3. Analytical Methods

MPA and fMPA concentrations were determined in the Department of Physical Pharmacy and Pharmacokinetics at Poznan University of Medical Sciences, Poland.

MPA plasma concentrations were determined using the HPLC method with ultraviolet detection. The analytical method for MPA determination was described elsewhere [11,57]. The calibration curve was linear, and within the range 0.25–40.0 μg/mL. The mean between-day coefficient of variation and average accuracy were 2.7% (range 0.5–6.1%) and 98.8% (range 93.8–103.0%), respectively [11].

Free MPA (fMPA) was determined using the HPLC method with fluorescence detection described previously [5,11]. The calibration curve was linear, and within the range of 0.0025–1.0 μg/mL. The mean between-day coefficient of variation and average accuracy were 6.5% (range 1.4–12.7%) and 99.9% (range 94.3–107.6%), respectively [11].

### 4.4. The Literature Data Search

We comprehensively searched the literature in December 2020 using the PUBMED database using the combination of ‘mycophenolic acid’ or ‘mycophenolate mofetil’ and the terms: ‘limited sampling strategy’, ‘limited sampling strategies’, ‘limited sampling’, ‘optimal sampling’, ‘sparse sampling’, and ‘minimal sampling’. We included English written studies determining LSS based on MLR calculations for adult and pediatric patients receiving MMF after solid organ transplantation or with autoimmune diseases, and identified those LSSs which covered the same blood sampling times as in our study. We included LSSs which were established based on HPLC and EMIT MPA determinations. We excluded articles describing LSS for EC-MPS as there is an evident difference in MPA pharmacokinetics for the two formulations MMF and EC-MPS (unpredictable absorption profile after EC-MPS administration) [58]. We also excluded studies using previously established LSSs, those with Bayesian estimators and with different than twice daily MMF dosing schedules.

### 4.5. Pharmacokinetic Calculations and Statistical Analyses

For children with nephrotic syndrome, firstly, we calculated the MPA AUC_total_ using the linear trapezoidal rule. Secondly, based on the results of the literature data search, we calculated the AUC_pred_ for these children using the MLR formulae found in the literature. We applied LSSs established using MPA concentrations determined with HPLC, EMIT, and PETINIA to evaluate LSS usefulness. Due to the 15–20% higher MPA concentrations established with EMIT [16] and the similar magnitude of the MPA overestimation found for PETINIA when compared with EMIT [53], we multiplied the MPA concentration determined in the children included in this study with the HPLC method by 1.175, and applied the re-calculated AUC_total_ to the evaluation of the LSSs based on EMIT or PETINIA MPA determination. The multiplier of 1.175 was achieved by assuming that MPA concentrations established with EMIT are on average 17.5% higher than those determined with HPLC.

To assess the predictive performance of LSSs available in the literature, we calculated r^2^ as well as the bias and precision for AUC_pred_ as the mean relative prediction error (%MPE) and the percentage of the mean absolute relative prediction error (%MAE), respectively, both with 95% confidence intervals. According to the literature, precision and bias ±15% were considered acceptable [22,59,60], although some authors defined the clinical acceptance as ±20% [18] or even as ±33% [61]. Although it does not translate into clinical practice, lower percentages of precision and bias result in more accurate calculations. We also calculated the percentage of the AUC_pred_ within ±15% of the AUC_total_ for each equation to analyze the agreement between the AUC_pred_ and the AUC_total_. The equations used in the analysis were as follows [51,62]:

(1) $$\%\mathrm{MPE}=\frac{1}{N}\Sigma \frac{({\mathrm{AUC}}_{\mathrm{pred}}-{\mathrm{AUC}}_{\mathrm{total})}}{{\mathrm{AUC}}_{\mathrm{total}}}\times 100$$

(2) $$\mathrm{MAE}=\frac{1}{N}\Sigma \frac{\left|{\mathrm{AUC}}_{\mathrm{pred}}-{\mathrm{AUC}}_{\mathrm{total}}\right|}{{\mathrm{AUC}}_{\mathrm{total}}}\times 100$$

Statistical analyses were performed using STATISTICA 13.0 software (StatSoft, Inc., Tulsa, OK, USA). For the best matched MLR LSSs, the Bland–Altman method was used to assess the agreement between the AUC_pred_ and the AUC_total_. To compare the HPLC and EMIT/PETINIA predictive performance results, the Mann–Whitney test was applied.

## 5. Conclusions

We concluded that the optimal MPA LSS for children with nephrotic syndrome should include C_1_, C_2,_ and C_6_, as these time points coincide with MPA C_max_ and C_max2_. MPA LSSs established using MPA concentrations determined with EMIT or PETINIA may be used in LSSs based on HPLC-determined MPA concentrations after multiplying the latter by 1.175. The MLR LSS which predicted MPA AUC the best in children with nephrotic syndrome was developed for MMF-treated renal transplant recipients. MPA binding with plasma protein is high in children with nephrotic syndrome, which suggests there are different fMPA pharmacokinetics in this group of patients than in renal, liver, and hematopoietic stem cell recipients treated with MMF. MPA LSSs may facilitate TDM in the case of MMF, however, more studies of fMPA LSS are required for children with nephrotic syndrome.

## Figures and Tables

**Table 1:** Plasma concentrations and exposure of MPA and fMPA in children with nephrotic syndrome.

	Parameter	Mean ± SD	Range
MPA	C_max_ (μg/mL)	18.20 ± 9.34	4.96–44.22
	t_max_ (h)	1 ± 1	1–3
	AUC_total_ (μg∙h/mL)	53.14 ± 17.77	22.27–94.54
fMPA	C_max_ (μg/mL)	0.0660 ± 0.0081	0.1605–0.0409
	AUC_total_ (μg∙h/mL)	0.1837 ± 0.0867	0.0551–0.3806

MPA, mycophenolic acid; fMPA, free mycophenolic acid; AUC_total_, area under the time–concentration curve from 0 to 12 h; SD, standard deviation.

**Table 2:** Predictive performance of MLR-based HPLC–MPA LSSs available in the literature for estimation of MPA AUC_pred_ in children with nephrotic syndrome treated with MMF.

No	Equation	Indication for MMF Treatment	Drugs Co-Administered	Reference	%MPE	%MAE	r^2^	% of AUC_pred_ within ±15% of AUC_total_
					(95% CI)	(95% CI)		
1	AUC_pred_ = 9.328 + 1.311 × C_1_ + 1.455 × C_2_ + 2.901 × C_4_	adult renal Tx	Tac	[43]	−0.55	11.68	0.807	67
					(−6.89–5.78)	(7.83–15.53)		
2	AUC_pred_ = 15.94 + 1.77 × C_2_ + 2.34 × C_4_ + 4.76 × C_9_	adult renal Tx	Tac, steroids	[41]	−5.08	15.74	0.619	50
					(−13.08–2.92)	(10.97–20.51)		
3	AUC_pred_ = 20.38 + 0.26 × C_0_ + 2.06 × C_2_ + 3.82 × C_4_	adult renal Tx	Tac, steroids	[41]	−4.19	17.23	0.465	46
					(−13.17–4.78)	(11.87–22.58)		
4	AUC_pred_ = 9.02 + 3.77 × C_0_ + 1.33 × C_1_ + 1.68 × C_3_ + 2.96 × C_6_	adult renal Tx	CsA, steroids	[37]	12.91	18.00	0.773	54
					(5.53–20.30)	(12.97–23.03)		
5	AUC_pred_ = 6.02 + 5.61 × C_0_ + 1.28 × C_1_ + 0.9 × C_2_ + 2.54 × C_4_	adult renal Tx	CsA, steroids	[48]	13.43	18.35	0.723	50
					(1.59–25.28)	(9.55–27.14)		
6	AUC_pred_ = 3.504 + 1.098 × C_1_ + 0.670 × C_2_ + 5.659 × C_4_	adult renal Tx	CsA, steroids	[36]	−14.12	19.95	0.684	33
					(−21.65–[−6.58])	(15.50–24.40)		
7	AUC_pred_ = 15.19 + 6.92 × C_0_ + 1.08 × C_1_ + 0.72 × C_2_	adult renal Tx	CsA, steroids	[48]	16.28	24.56	0.527	42
					(4.44–28.13)	(15.76–33.36)		
8	AUC_pred_ = −0.247 + 11.73 × C_6_ + 2.92 × C_2_	adult renal Tx	CsA, steroids	[39]	3.04	26.45	0.487	46
					(−11.63–17.71)	(17.13–35.76)		
9	AUC_pred_ = 9.57 × C_6_ + 27.238	adult renal Tx	no data	[38]	9.88	28.31	0.265	29
					(−4.88–24.63)	(18.99–37.62)		
10	AUC_pred_ = 10.403 + 0.841 × C_2_ + 1.105 × C_3_ + 0.447 × C_4_	adult renal Tx	CsA, steroids	[36]	−54.94	54.94	0.372	0
					(−59.83–[−50.05])	(50.05–59.83)		
11	AUC_pred_ = 10.229 + 0.925 × C_1_ + 1.750 × C_2_ + 4.586 × C_6_	adult liver Tx	Tac, steroids	[29]	0.49	12.57	0.823	63
					(−6.08–7.05)	(8.85–16.28)		
12	AUC_pred_ = 17.930 + 1.992 × C_2_ + 4.136 × C_6_	adult liver Tx	Tac, steroids	[29]	−12.17	18.22	0.565	50
					(−20.08–[−4.25])	(12.89–23.54)		
13	AUC_pred_ = 1.783 + 1.248 × C_1_ + 0.888 × C_2_ + 8.027 × C_4_	adult islet Tx	Tac	[22]	4.18	17.47	0.648	50
					(−6.31–14.68)	(9.94–24.99)		
14	AUC_pred_ = 2.778 + 1.413 × C_1_ + 0.963 × C_3_ + 7.511 × C_4_	adult islet Tx	Tac	[22]	4.04	17.93	0.619	50
					(−6.34–14.41)	(10.80–25.06)		
15	AUC_pred_ = 1.547 + 1.417 × C_1_ + 9.448 × C_4_	adult islet Tx	Tac	[22]	5.48	21.29	0.557	50
					(−7.15–18.10)	(12.31–30.28)		
16	AUC_pred_ = 1.410 − 0.259 × C_0_ + 1.443 × C_1_ + 9.622 × C_4_	adult islet Tx	Tac	[22]	5.60	21.86	0.551	50
					(−4.78–15.97)	(14.73–28.99)		
17	logAUC_pred_ = 1.024 + 0.192 × logC_0_ + 0.213 × logC_1_ + 0.355 × logC_2_	adult lung Tx	CsA, steroids	[44]	−14.11	17.79	0.718	42
					(−20.76–[−7.45])	(13.05–22.53)		
18	logAUC_pred_ = 1.14 + 0.241 × logC_0_ + 0.406 × logC_2_	adult lung Tx	CsA, steroids	[44]	−25.96	28.88	0.427	21
					(−34.21–[−17.72])	(22.70–35.07)		
19	AUC_pred_ = 4.43 + 2.76 × C_0_ + 0.51 × C_1_ + 1.97 × C_2_ + 4.27 × C_6_	adult HSCT	CsA	[42]	−8.34	15.79	0.708	54
					(−15.19–[−1.50])	(12.13–19.45)		
20	AUC_pred_ = 1.2039 × AUC_1–4_ + 8.9727	adult HSCT	CsA	[34]	−31.85	31.85	0.841	4
					(−35.91–[−27.80])	(27.80–35.91)		
21	AUC_pred_ = 0.10 + 11.15 × C_0_ + 0.42 × C_1_ + 2.80 × C_2_	adult heart Tx	CsA, steroids	[45]	15.24	31.94	0.366	33
					(−1.66–32.14)	(20.15–43.72)		
22	AUC_pred_ = −0.51 + 11.47 × C_0_ + 3.24 × C_2_	adult heart Tx	CsA, steroids	[45]	8.19	35.54	0.264	25
					(−10.63–27.02)	(24.06–47.02)		
23	AUC_pred_ = 13.81 + 0.68 × C_1_ + 1.08 × C_2_ + 2.21 × C_3_ + 4.62 × C_0_	children systemiclupus erythematosus	none	[21]	9.82	16.26	0.738	50
					(1.38–18.25)	(9.95–22.57)		

AUC_pred_, predicted area under the time(0–12 h)–concentration curve; AUC_total_, determined area under the concentration—time curve from 0 to 12 h; CI, confidence interval; CsA, cyclosporine; HPLC, high performance liquid chromatography; HSCT, hematopoietic stem cell transplantation; LSSs, limited sampling strategies; MMF, mycophenolate mofetil; MLR, multiple linear regression; MPA, mycophenolic acid; %MAE, percentage of mean absolute relative prediction error; %MPE, mean relative prediction error; Tac, tacrolimus; Tx, transplantation.

**Table 3:** The predictive performance of MLR-based EMIT/PETINIA-MPA LSSs available in the literature for estimation of MPA AUC_pred_ in children with nephrotic syndrome treated with MMF.

No	Equation	Indication for MMF Treatment	Drugs Co-Administered	Reference	%MPE	%MAE	r^2^	% of AUC_pred_ within ±15% of AUC_total_
					(95% CI)	(95% CI)		
1	AUC_pred_ = 10.6 + 1.1 × C_1_ + 1.1 × C_2_ + 2.0 × C_4_ + 3.9 × C_6_	adult renal Tx	Tac, steroids	[30] ^1^	2.90	11.56	0.860	67
					(−2.92–8.73)	(8.30–14.82)		
2	AUC_pred_ = 7.4 + 2.3 × C_0_ + 1.2 × C_1_ + 2.3 × C_3_ + 4.4 × C_6_	adult renal Tx	Tac, steroids	[30] ^1^	7.32	12.21	0.829	71
					(1.50–13.14)	(8.20–16.22)		
3	AUC_pred_ = 3.8 + 3.5 × C_0_ + 1.2 × C_1_ + 1.9 × C_3_ + 5.4 × C_6_	adult renal Tx	Tac, steroids	[30] ^1^	9.85	15.90	0.742	63
					(2.24–17.47)	(10.51–21.28)		
4	AUC_pred_ = 4.42 + 1.74 × C_1_ + 2.99 × C_4_ + 5.43 × C_9_	adult renal Tx	CsA	[40]	8.16	15.92	0.826	58
					(0.88–15.43)	(11.67–20.18)		
5	AUC_pred_ = 17.3 + 4.4 × C_0_ + 1.1 × C_1_ + 2.9 × C_4_	adult renal Tx	Tac, steroids	[27]	9.13	18.63	0.638	50
					(0.09–18.17)	(12.91–24.35)		
6	AUC_pred_ = 23.37 + 4.21 × C_0_ + 3.60 × C_4_	adult renal Tx	Tac	[47]	−12.35	21.82	0.198	46
					(−22.77–[−1.92])	(14.85–28.78)		
7	AUC_pred_ = 4.38 + 2.14 × C_1_ + 7.19 × C_9_	adult renal Tx	CsA	[40]	11.62	22.50	0.722	42
					(0.49–22.75)	(15.10–29.91)		
8	AUC_pred_ = 20.30 + 5.80 × C_0_ + 3.06 × C_4_	adult renal Tx	Tac	[47]	−12.12	23.57	0.160	42
					(−25.13–0.88)	(18.18–28.96)		
9	AUC_pred_ = 8.149 + 1.442 × C_2_ + 1.056 × C_4_ + 7.133 × C_6_	adult renal Tx	Tac, steroids	[26]	−20.52	25.56	0.501	25
					(−29.19–[−11.85])	(19.90–31.21)		
10	AUC_pred_ = 22.93 + 4.63 × C_0_ + 5.60 × C_6_	adult renal Tx	Tac	[47]	−1.86	27.50	0.208	17
					(−14.87–11.14)	(22.11–32.89)		
11	AUC_pred_ = 14.9 + 1.3 × C_1_ + 3 × C_4_ + 3.7 × C_6_	adult renal Tx	Tac, steroids	[27]	96.25	98.30	0.549	4
					(71.31–121.19)	(74.90–121.71)		
12	AUC_pred_ = 5.92 + 1.10 × C_1_ + 1.01 × C_2_ + 1.77 × C_4_ + 4.80 × C_6_	adult liver Tx	Tac, steroids	[28]	−3.29	11.84	0.829	67
					(−9.47–2.88)	(8.09–15.59)		
13	AUC_pred_ = 8.144 + 2.880 × C_3_	adult liver Tx	Tac, steroids	[31]	−62.44	62.44	0.134	0
					(−68.53–[−56.35])	(56.35–68.53)		
14	AUC_pred_ = 8.22 + 3.16 × C_0_ + 0.99 × C_1_ + 1.33 × C_2_ + 4.18 × C_4_	children renal Tx	CsA	[32]	7.93	12.58	0.799	67
					(1.47–14.39)	(7.68–17.48)		
15	AUC_pred_ = 8.217 + 3.163 × C_0_+ 0.994 × C_1_ + 1.334 × C_2_ + 4.183 × C_4_	children renal Tx	CsA	[8]	8.14	12.65	0.799	67
					(1.68–14.61)	(7.71–17.58)		
16	AUC_pred_ = 7.73 + 0.94 × C_1_ + 2.55 × C_2_ + 5.48 × C_6_	children renal Tx	CsA	[32]	8.94	14.67	0.829	58
					(2.19–15.68)	(10.17–19.18)		
17	AUC_pred_ = 10.75 + 0.98 × C_1_ + 2.38 × C_2_ + 4.86 × C_6_	children renal Tx	CsA	[33]	10.08	14.76	0.842	50
					(3.46–16.66)	(10.10–19.42)		
18	AUC_pred_ = 12.62 + 7.78 × C_0_ + 0.9 × C_1_ + 1.3 × C_2_	children renal Tx	CsA	[9]	13.81	23.20	0.515	50
					(2.00–25.62)	(14.55–31.85)		
19	AUC_pred_ = 13.73 + 9.024 × C_0_ + 1.779 × C_2_	children renal Tx	CsA	[9]	0.31	28.79	0.203	21
					(−14.71–15.34)	(20.33–37.25)		
20	AUC_pred_ = 15.1 + 9.68 × C_0_ + 1.28 × C_1_	children renal Tx	CsA	[9]	23.57	33.21	0.374	29
					(8.22–38.91)	(21.65–44.77)		
21	AUC_pred_ = 12.3 + 4.7 × C_0_ + 1.2 × C_1_ + 2.7 × C_3_ + 1.8 × C_6_	adult autoimmune disease	CsA	[46]	18.85	20.15	0.811	50
					(11.45–26.25)	(13.42–26.88)		
22	AUC_pred_ = 17.5 + 7.1 × C_0_ + 1.0 × C_1_ + 2.6 × C_3_	adult autoimmune disease	CsA	[46]	24.84	27.45	0.607	33
					(13.36–36.02)	(17.47–37.43)		
23	AUC_pred_ = 38.3 + 11.7 × C_0_	adult autoimmune disease	CsA	[46]	35.64	47.39	0.051	21
					(13.52–57.76)	(29.84–64.94)		
24	AUC_pred_ = 21.971 + 2.6059 × C_2_	children INS	CsA	[14] ^1^	−24.57	26.14	0.455	33
					(−32.54–[−16.59])	(19.16–33.12)		
25	AUC_pred_ = 8.7 + 4.63 × C_0_ + 1.90 × C_1_ + 1.52 × C_2_	children NS	none	[25]	24.21	29.03	0.718	17
					(14.28–34.13)	(21.90–36.15)		

AUC_pred_, predicted area under the (0–12 h) time–concentration curve; AUC_total_, determined area under the concentration—time curve from 0 to 12 h; CI, confidence interval; CsA, cyclosporine; EMIT, enzyme multiplied immunoassay technique; INS, idiopathic nephrotic syndrome; LSSs, limited sampling strategies; MMF, mycophenolate mofetil; MLR, multiple linear regression; MPA, mycophenolic acid; %MAE, percentage of mean absolute relative prediction error; %MPE, mean relative prediction error; NS, nephrotic syndrome; PETINIA, particle enhanced turbidimetric inhibition immunoassay; Tac, tacrolimus; Tx, transplantation. ^1^ MPA determined with particle enhanced turbidimetric inhibition immunoassay (PETINIA).

**Table 4:** The predictive performance of MLR-based HPLC-fMPA LSSs available in the literature for the estimation of fMPA AUC_pred_ in children with nephrotic syndrome treated with MMF.

No	Equation	Indication for MMF Treatment	Drugs Co-Administered	Reference	%MPE	%MAE	r^2^	% of AUC_pred_ within ±15% of AUC_total_
					(95% CI)	(95% CI)		
1	fMPA AUC_pred_ = 34.2 + 1.12 × C_1_ + 1.29 × C_2_ + 2.28 × C_4_ + 3.95 × C_6_	liver Tx	Tac, steroids	[35]	13.68	18.53	0.871	38
					(6.44–20.91)	(13.71–23.35)		
2	fMPA AUC_pred_ = 63.92 + 2.01 × C_0_ + 0.67 × C_1_ + 2.05 × C_2_ + 4.26 × C_6_	HSCT	CsA	[42]	−14.45	22.17	0.725	33
					(−23.61–[−5.28])	(16.56–27.77)		
3	fMPA AUC_pred_ = 136.826 + 0.76 × C_1_ + 0.84 × C_2_ + 3.914 × C_4_	renal Tx	CsA, steroids	[36]	52.65	54.69	0.768	21
					(29.91–75.39)	(32.86–76.52)		
4	fMPA AUC_pred_ = 178.167 + 0.954 × C_2_ + 4.001 × C_4_	renal Tx	CsA, steroids	[36]	59.46	63.35	0.564	43
					(28.68–90.25)	(34.04–92.65)		
5	fMPA AUC_pred_ = 180.543 + 0.956 × C_2_ − 0.223 × C_3_ + 4.342 × C_4_	renal Tx	CsA, steroids	[36]	61.48	64.84	0.560	25
					(30.43–92.54)	(35.08–94.60)		

AUC_pred_, predicted area under the (0–12 h) time–concentration curve; AUC_total_, determined area under the concentration—time curve from 0 to 12 h; CI, confidence interval; CsA, cyclosporine; fMPA, free mycophenolic acid; LSSs, limited sampling strategies; HSCT, hematopoietic stem cell transplantation; MMF, mycophenolate mofetil; MLR, multiple linear regression; %MAE, percentage of mean absolute relative prediction error; %MPE, mean relative prediction error; Tac, tacrolimus; Tx, transplantation.

**Table 5:** Demographic and biochemical characteristics of the study group.

Parameter	Mean ± SD	Range
24 children	10 boys/14 girls	
age	11 ± 4	3–18
body weight	36.9 ± 14.7	17.7–66.5
body surface (m^2^)	1.20 ± 0.32	0.70–1.85
MMF daily dose (mg)	Number of children	
500/600/700/800/1000/1200/1500/2000	2/1/1/1/10/1/7/1	
MMF daily dose (mg/m^2^)	933 ± 218	505–1250
duration of MMF treatment (months)	12 ± 7	2–29
Protein concentration (g/dL)	6.60 ± 0.53	5.52–7.54
Glomerular filtration rate (mL/min/1.73 m^2^)	133 ± 23	101–183
Creatinine concentration (mg/dL)	0.45 ± 0.13	0.25–0.72
Leukocytes count (10^9^/L)	6.75 ± 2.34	3.46–13.88
Erythrocytes count (10^12^/L)	4.65 ± 0.31	4.07–5.54
Hemoglobin (g/dL)	13.0 ± 1.1	11.1–15.5
Hematocrit (%)	37.8 ± 2.8	33.6–44.3
Alanine aminotransferase (U/L)	13 ± 4	5–25
Aspartate aminotransferase (U/L)	26 ± 6	17–45

MMF, mycophenolate mofetil; SD, standard deviation.

## Data Availability

The data presented in this study are available on request from the corresponding author.
